# Summary Outcomes of the ODIN Project on Food Fortification for Vitamin D Deficiency Prevention

**DOI:** 10.3390/ijerph15112342

**Published:** 2018-10-24

**Authors:** Mairead Kiely, Kevin D. Cashman

**Affiliations:** Cork Centre for Vitamin D and Nutrition Research, School of Food and Nutritional Sciences, University College Cork, T12ND89 Cork, Ireland; k.cashman@ucc.ie

**Keywords:** vitamin D, 25-hydroxyvitamin D, food fortification, dietary requirements, dietary modelling, bio-fortification

## Abstract

Food-based solutions for optimal vitamin D nutrition and health through the life cycle (ODIN) was a cross-disciplinary, collaborative project, including 30 partners from 19 countries, which aimed to develop evidence-based solutions to prevent low vitamin D status (25-hydroxyvitamin D (25(OH)D) < 30 nmol/L) using a food-first approach. This paper provides a summary overview of some of the important ODIN outcomes and outlines some outstanding data requirements. In a study of almost 56,000 individuals, the first internationally standardised dataset of vitamin D status showed that 13% of EU residents overall, across a latitude gradient of 35° N to 69° N, had serum 25(OH)D < 30 nmol/L and 40% were < 50 nmol/L. The risk of low vitamin D status was several-fold higher among persons of ethnic minority. However, additional data from quality bio-banked sera would be required to improve these estimates. To address the question of dietary requirements for vitamin D among under-researched life-stage and population groups, four dose-response RCTs conducted in Northern Europe showed that vitamin D_3_ intakes of 8 and 13 μg/day prevented 25(OH)D decreasing below 30 nmol/L in white children and adolescents and 20 and 30 μg/day, respectively, achieved ≥50 nmol/L. Among white women during pregnancy, 30 μg/day is required to prevent umbilical cord 25(OH)D, representing new-born vitamin D status, below 25 nmol/L. While 8 μg/day protected white women in Finland at the 30 nmol/L cut-off, 18 μg/day was needed by women of East African descent to prevent 25(OH)D decreasing below 30 nmol/L during wintertime. Replicate RCTs are needed in young children <5 years and in school-age children, teens and pregnant women of ethnic minority. Using a series of food production studies, food-based RCTs and dietary modelling experiments, ODIN research shows that diverse fortification strategies could safely increase population intakes and prevent low vitamin D status. Building on this solid technological platform, implementation research is now warranted to scale up interventions in real-world settings to eradicate vitamin D deficiency.

## 1. Introduction

To prevent nutritional rickets and osteomalacia, which have severe and lasting consequences for bone growth and skeletal integrity throughout life, it is generally agreed that prevention of vitamin D deficiency and low vitamin D status is a public health priority. Non-skeletal health effects of low vitamin D status, including infection, cancer, perinatal health outcomes and cardiovascular disease are the subject of a global research effort. A comprehensive systematic literature review (SLR) and meta-analysis from Autier et al. [1] identified common upper respiratory tract infections and asthma exacerbations as the most promising new findings from recent randomized controlled trials (RCTs) and SLRs of RCTs. No positive signals on other health outcomes had emerged up to then, further strengthening the view that rather than causing ill-health, low vitamin D status may be its consequence, driving observations in epidemiological studies through reverse causation [2]. The vitamin D community awaits imminent results from several mega-trials, described by Ebeling and colleagues [2], including VITamin D and OmegA-3 TriaL (VITAL), which has recruited almost 26,000 participants, of whom ~17,000 have provided a baseline blood sample. Primary outcomes are expected in late 2018 [3,4]. Should VITAL and other ongoing vitamin D RCTs demonstrate evidence for non-skeletal health effects of higher circulating 25-hydroxyvitamin D (25(OH)D) (in excess of the 50 nmol/L for bone health), re-evaluations of the nutritional requirements for vitamin D will be prompted, leading to revised dietary recommendations. In the meantime, current dietary recommendations for individuals, based on bone health outcomes, vary between 10 μg (400 IU) and 20 μg (800 IU)/day, depending on whether the target 25(OH)D is 25/30 or 50 nmol/L [5,6,7,8,9,10], reviewed in detail [11,12].

Notwithstanding debate on the optimal target 25(OH)D concentration, the context of this article is that in Europe, low vitamin D status (with 25(OH)D concentrations <30 nmol/L) is still relatively common, which is unacceptable given that a vitamin D intake of ~10 μg/day prevents circulating 25(OH)D from dropping below this minimum threshold [5,6]. Thus, this overview of recent data from Europe will describe dietary strategies, namely food fortification and bio-fortification, to bridge the gap between current dietary intakes of vitamin D and the intakes that are required to prevent low vitamin D status, as well as some new data to inform dietary requirements among under-researched groups.

## 2. The ODIN Project

Food-based solutions for optimal vitamin D nutrition and health through the life cycle (ODIN) was a large scale collaborative project, funded by the European Commission, which kicked off in November 2013. Jointly co-ordinated by the authors, ODIN was a trans- and multidisciplinary consortium of 30 partners, including analytical chemists, food scientists, data analysts, geneticists, epidemiologists, endocrinologists, paediatricians, physicists and nutrition scientists, from 19 countries, including Ireland (lead), the United Kingdom, Spain, Portugal, Norway, Denmark, Iceland, Finland, Austria, Germany, Slovakia, Serbia, Switzerland, Greece, the United States, Ukraine, Belgium, the Netherlands and Canada. Built around the research recommendations from a detailed analysis provided by Cashman and Kiely in 2011 [11] of persistent knowledge gaps in vitamin D nutrition and public health, ODIN used its budget of 6M euro to address a series of priorities, described on behalf of the consortium by Kiely and Cashman [13].

The aim of the ODIN project was to develop effective, safe and sustainable solutions to prevent vitamin D deficiency and improve vitamin D-related health outcomes using a food-first approach. Bearing in mind our conservative budget, the project selected the most critical nutrition-related issues in the vitamin D field for attention, clustering them around knowledge gaps and ensuring data flow to maximise efficiency in the project. Questions addressed were under the broad headings of vitamin D status, UVB and dietary exposure, dietary recommendations, dietary modelling and health and safety effects. Underpinning data to achieve these outcomes were delivered by the consortium across an ambitious programme encompassing both dose-response and food-based RCTs, research in primary food production and food technology, data mining of epidemiological cohort studies and dietary modelling experiments. The quality and reliability of ODIN analytical data, both serum 25(OH)D and related metabolites, as well as food composition data, was a cornerstone of excellence in the project, assuring validity of experimental outcomes and providing reference data for discrete population groups. This paper provides a summary overview of some of the significant ODIN outcomes that have been published over the last three years from the priority research around vitamin D status, dietary intakes, dietary recommendations for vitamin D and fortification strategies to increase vitamin D in the food supply (see Box 1). Other packages of work not described here due to space constraints were focussed on vitamin D and health in older adults, perinatal health, safety of chronic exposure to high vitamin D intakes and status, UVB availability and the role of sun exposure.

Box 1 **Selected ODIN questions.**

1. What is the prevalence of vitamin D deficiency in Europe?
2. What are the dietary requirements for vitamin D during pregnancy, childhood and adolescence to prevent vitamin D deficiency?
3. Does ethnicity affect dietary requirements for vitamin D?
4. Can fortification/biofortification increase population intakes of vitamin D by enough to reduce the prevalence of inadequate intakes safely?

## 3. Results

### 3.1. What is the Prevalence of Vitamin D Deficiency in Europe?

In 2016, ODIN delivered the first internationally standardised dataset of vitamin D status and reported the prevalence of vitamin D deficiency across Europe for the first time, enabling within and between-country comparisons. While there was considerable variation across study populations, 13% overall, across a latitude gradient of 35° N to 69° N, had serum 25(OH)D < 30 nmol/L [14]. These prevalence rates are roughly twice those reported in the United States (5.9%) [15] and Canada (7.4%) [16] and translate into enormous numbers of individuals. Based on recent census data, ODIN deficiency estimates of 12.5–15.2%, 12.3% and 22% in the nationally representative nutrition and health surveys for Germany, Ireland and the UK, respectively, amount to >25 million individuals with 25(OH)D <30 nmol/L in these member states alone. Relative to the 25(OH)D threshold of 50 nmol/L for protection of skeletal and non-skeletal health [10], Cashman et al. [14] reported a prevalence of 40% below 50 nmol/L. Thus, on the basis of the World Health Organization’s criteria [17] and the 25(OH)D threshold (30 or 50 nmol/L) selected, vitamin D deficiency in Europe can be classified as a mild (5–19.9%) or severe (>40%) public health problem.

Cashman et al. [14] described how ODIN applied the protocols developed by the NIH-led Vitamin D Standardization Program (VDSP) [18,19] to existing serum 25(OH)D data from 18 nationally and regionally representative studies of children, teens, adults and elderly individuals (*n* = 55,844), achieving geographical coverage of the European region. Using the Cork Centre for Vitamin D and Nutrition Research CDC-certified LC-MS/MS platform to perform 25(OH)D re-analysis of uniformly selected subsamples from each population, the VDSP approach allowed ODIN to generate reliable prevalence estimates of vitamin D deficiency in Europe using standardized serum 25(OH)D data. These are the first internationally standardised prevalence estimates of vitamin D status and provided firm evidence that deficiency is widespread across Europe, at rates indicative of a serious public health problem, underlying not only metabolic bone diseases but potentially other health outcomes as well. The editorial that accompanied this paper [20] emphasised the need for standardisation of population-based 25(OH)D measurements using the VDSP protocols and cautioned against using laboratory analyses of variable quality.

**Figure 1:**
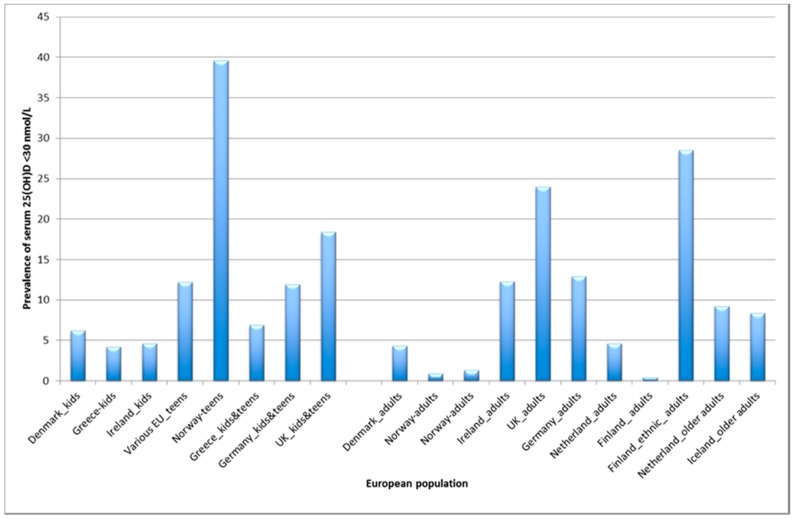
Prevalence of vitamin D deficiency (serum 25(OH)D < 30 nmol//L) in European young and adult/older adult, populations (adapted from Cashman et al [14]).

As well as quantifying the size of the vitamin D deficiency problem in Europe, these data, stratified by country and population sub-group, identified those most at risk. The data in Figure 1 highlight a relatively high risk of very low vitamin D status (<30 nmol/L) among adolescents (between 18 and 40% in the UK and Norway) compared with young children (<5% in Ireland and Greece). Notably, persons of ethnic minority were at much higher risk than their white counterparts. Compared to Whites in the UK, Norway and Finland, data from ethnic minorities showed a far higher yearly prevalence of vitamin D deficiency [14], with 18 to 65% <30 nmol/L in those countries (http://www.odin-vitd.eu/public/7-european-vitamin-d-deficiency-map/). In the Finnish Migrant Health and Well-being Study (Maamu; *n* 1310), which is representative of immigrant populations in Finnish cities, the prevalence of serum 25(OH)D < 30 nmol/L was 4.5, 28.0 and 50.4% for white Russian-speaking, Somali and Kurdish (born in Iran or Iraq) subgroups, respectively. Albeit with small numbers, the UK National Diet and Nutrition Survey (NDNS years 1–4) showed that 35.7% of Black and 59.6% of Asian participants had 25(OH)D < 30 nmol/L, compared with 19.6% of Whites. As the proportion of participants from ethnic minorities was low in most other studies in the ODIN exercise, it was not appropriate to report stratified data, but in all cases, estimates for non-white participants were higher compared with equivalently-aged white individuals within a study population [14]. One of the most disturbing outcomes from the work was the lack of well-curated and characterised biobanks of ethnic minorities resident in Europe; this is a massive research gap that must be prioritised to identify needs and avoid persistent health inequalities.

Providing data for regions less comprehensively surveyed was a challenge in the absence of available biobanks and survey databases accessible to the consortium. Therefore, ODIN scientists conducted systematic literature reviews of available data on serum 25(OH)D concentrations in Southern European and Eastern Mediterranean countries as well as vitamin D intakes and status in Central and Eastern European countries. From their analysis of 107 studies, stratified by sex and age group, representing 630,093 individuals, Manios and colleagues [21] reported that more than one-third of studies had mean 25(OH)D concentrations < 50 nmol/L and about 10% reported mean serum 25(OH)D < 25 nmol/L. Infants and elderly adults were the most susceptible age groups for low vitamin D status among Southern and Eastern Mediterranean countries. Many countries in the Central and Eastern European region, including Albania, Belarus, Bosnia and Herzegovina, Bulgaria, Latvia, Macedonia, Moldova, Montenegro, and Slovakia had no published data on vitamin D intakes or status. These data have highlighted the need for strategic investment in quality, accessible surveillance and bio-banking systems among many countries in the Mediterranean, Central and Eastern European regions.

### 3.2. Dietary Requirements for Vitamin D: Children, Adolescents, Pregnant Women and Persons of Ethnic Minority

One of our core commitments in the ODIN project was to address under-represented groups among the healthy population for whom data to base estimates for vitamin D recommendations were scarce or absent [5,6,7,8,9,10]; namely young children, adolescents, pregnant women and persons of ethnic minority. Thus, four 25(OH)D-vitamin D_3_ dose-response RCTs were designed and implemented to estimate the dietary requirements for vitamin D to meet 25(OH)D targets of 25/30 to 50 nmol/L in most (97.5%) individuals in these subgroups, under conditions of absent or scarce UVB exposure, providing evidence on which to base dietary allowances for them [22,23,24,25,26].

#### 3.2.1. Children and Adolescents

Dietary requirements for vitamin D during childhood and adolescence have been predominantly based on two studies in 8 and 11 year olds (summarised in [5]), leaving large gaps among younger children and teens. Accordingly, two parallel, dose-response RCTs in Denmark (55° N) and the UK (51° N) were implemented during winter months to establish the distribution of dietary requirements for vitamin D in 4–8 year olds and 14–18 year olds in the absence of UVB exposure [22,23]. Children and teens were randomised to placebo, 10 or 20 μg/day of vitamin D_3_ from October through March. Vacation in a sunny location and sun-bed use were exclusion criteria.

Briefly, the outcomes of these RCTS were:

(1) For white 4–8 year-old children, 8 µg/day of vitamin D_3_ prevented 25(OH)D falling below 30 nmol/L during wintertime and 20 µg/day maintained 97.5% ≥50 nmol/L [22];
(2) For white 14–18 year-old adolescents, intakes of 13 µg/day of vitamin D_3_ were needed to prevent 25(OH)D from dropping <30 nmol/L and ~30 µg/day was required to achieve ≥50 nmol/L during winter [23].

It should be noted that in a subsequent unrelated study in Northern and Southern Sweden, at 63 and 55° N [27], white and dark-skinned children (aged 5–7 years) had varying requirements for vitamin D intakes, needing 6 and 14 μg/day, respectively to maintain 25(OH)D > 30 nmol/L, and 20 and 28 μg/d, respectively, to reach ≥50 nmol/L during winter. This suggests a variable dose-response to vitamin D_3_ between persons of different ethnic backgrounds.

#### 3.2.2. Women of Ethnic Minority in Northern Europe

In a third ODIN dose-response RCT with a similar design to those in children and adolescents, vitamin D requirements of 69 women of Finnish descent were compared with 47 East African women in Helsinki (60° N), during wintertime [24,25]. By way of context for this RCT, Finland has a mandatory fortification programme of fluid milk products and spreads and there is a high level of awareness of vitamin D nutrition and health among Finnish residents of all nationalities [24]. At the baseline assessment, there were some differences between Finnish and Somali women; on average, Somali participants were older (43 vs. 31 years), heavier (BMI of 28.9 vs. 23.2), had a higher vitamin D intake from a combination of diet and supplements (22.5 vs. 14.1 μg/day) and a lower 25(OH)D (52.2 vs. 60.5 nmol/L). Following a dose-response intervention of 0, 10 or 20 μg/day of vitamin D_3,_ models adjusted for baseline differences found that dietary requirements for vitamin D to maintain wintertime 25(OH)D above 30 nmol/L were significantly higher in women of East African descent than in women of Finnish descent.

(3) For white women, 8 µg/day vitamin D_3_ prevented 25(OH)D falling <30 nmol/L in winter, but 18 µg/day was needed for women of East African descent to meet this minimum threshold [25].

In agreement with the study in Swedish children [27] and research in the US by Gallagher et al. [28] and others, this study adds further evidence that there are ethnic differences in this study adds further evidence that there are differences in the dietary requirements for vitamin D among people of various ethnic backgrounds. Many more discrete studies, stratified by ethnicity, are needed to target the needs of diverse populations. These studies will require validated underpinning data on dietary intakes and food sources of vitamin D among ethnic minorities. Insufficient clinical data among persons of ethnic minority is a function of the under-representation of minority groups in clinical research [29]; this presents a global challenge for development of evidence-based recommendations for vitamin D and has profound implications for policy makers, the public, the medical profession and the food industry.

#### 3.2.3. Pregnant Women and New-Born Infants

Pregnancy and infancy are life-stages for which evidence of low vitamin D status is widespread but the evidence basis for setting dietary requirements for vitamin D is weakest [5]. Currently, dietary recommendations for pregnant and lactating women are the same as for non-pregnant individuals, due to the absence of dose-response trial data on which to base pregnancy-specific guidelines [30,31]. Thus, there is a lack of evidence for pregnancy and the neonatal period across a number of criteria; not only are recommendations not specific for perinatal health outcomes, they do not consider fetal and neonatal requirements and rely on an assumption that pregnancy does not increase the metabolic demand for vitamin D [30]. At a minimum, maternal vitamin D deficiency should be prevented to safeguard fetal skeletal development [32]. However, due to the gap between maternal and fetal circulating 25(OH)D, prevention of maternal deficiency (at the 30 nmol/L cut-off) during pregnancy will not ensure fetal protection [33]. We proposed that vitamin D recommendations during pregnancy should be established from the perspective of the fetal requirement, which is unknown [26]. This signifies a paradigm shift in determining nutritional requirements during pregnancy.

Thus, to estimate the vitamin D intake required to maintain maternal 25(OH)D in late gestation at concentrations sufficient to prevent *neonatal* deficiency at about 25/30 nmol/L, we conducted a three-arm, dose-response, double-blind, RCT in Cork (51° N) among 144 white-skinned pregnant women [26]. The study showed that when maternal 25(OH)D was ≥ 50 nmol/L, 95% of cord sera were ≥30 nmol/L and 99% were >25 nmol/L. Using a curvilinear regression model, we predicted the total vitamin D intake (from diet, antenatal supplements plus treatment dose) that maintained maternal 25(OH)D at the concentration sufficient to keep cord 25(OH)D ≥ 25–30 nmol/L.

(4) Among white women, 30 μg/d vitamin D_3_ safely maintained serum 25(OH)D concentrations ≥50 nmol/L during pregnancy at ~50° N, which ensured that 25(OH)D was >25 nmol/L in 99% and ≥30 nmol/L in 95% of umbilical cord sera [26].

While increasing vitamin D in the food supply is important for preventing very low vitamin D status in the population, these data show that pregnant women and new-born infants, who should be under medical supervision, require dual targeted supplementation with vitamin D. Replicate studies are required among pregnant adolescents and women of ethnic minority.

In summary, for prevention of seasonal 25(OH)D decreases in Northern Europe below the minimum cut-off of 25/30 nmol/L, intakes of about 10 μg/day of vitamin D_3_ are required among white children and adolescents. Between 20 and 30 μg/day will protect women of East African descent at the 30 nmol/L threshold and will achieve 50 nmol/L among white children and teens. For protection of neonatal vitamin D status at 25/30 nmol/L among white women, maternal supplementation is required to ensure a total intake of 30 μg/day. According to most international guidelines ([32] and others), infants require 10 μg/day during the first year of life, at least.

To provide a composite estimate across the (white-skinned, non-pregnant/lactating) population over 5 years of age, we completed an individual participant data (IPD)-level meta-regression from seven suitable winter-based RCTs including 882 participants from 4 to 90 years [34]. Notably, vitamin D intakes and serum 25(OH)D concentrations had been analyzed using the same methods in these studies, ensuring compatibility across the dose-response relationship. The IPD analysis confirmed that 10, 13 and 26 µg/day would be required to maintain circulating 25(OH)D > 25, 30, and 50 nmol/L, respectively in almost all persons (i.e., at the level of a Recommended Dietary Allowance) [34].

### 3.3. Can Fortification Increase Population Intakes of Vitamin D by Enough to Reduce the Prevalence of Inadequate Intakes Safely?

#### 3.3.1. Vitamin D Intakes

As much of Europe experiences 4–8 months of winter during which UVB availability is too low to permit cutaneous synthesis of cholecalciferol [35], dietary intakes of vitamin D are critical for prevention of low vitamin D status. Natural sources of vitamin D_3_ and 25(OH)D include oily fish, meat, dairy and eggs and ergocalciferol (vitamin D_2_) is readily produced in mushrooms exposed to UVB (see [36] for a full description of vitamin D content of foods). Depending on legislation, some foods are fortified with vitamin D_3_, including milk, yoghurt, spreads, cheese, juices, breads and breakfast cereal, and some are naturally enhanced (bio-fortified), including milk, mushrooms, eggs, meat and bread (fully described in [37]). Rich vitamin D food sources are consumed infrequently (e.g., oily fish), but other, less rich sources, such as beef and eggs, make an important contribution to intakes because they are frequently consumed [36]. Habitual vitamin D intakes frequently fall well below the minimum dietary recommendation of 10 μg/day throughout Europe [38,39] and internationally [40,41]. Typical average intakes in the EU are generally around 3–7.5 μg, depending on the country (reviewed in [42]). Vinas et al. [38] showed that among European national nutrition surveys reporting vitamin D intake data from 2000 onwards, 77–100% and 55–100% of adults (19–64 years) and elderly (>64 years), respectively, had intakes below 10 μg/day. 

#### 3.3.2. Vitamin D-Specific Issues in Dietary Surveys

Apart from the usual variability around dietary collection methods, analysis and reporting, which complicates efforts to complete inter-country comparisons of vitamin D intakes from national dietary surveys, there are three very specific issues particular to vitamin D that must be considered when reporting or analysing food consumption/dietary data; the food composition database, its provenance and quality; the country-specific fortification practices and regulations and the usual intakes of dietary supplements containing vitamin D. Surveys that do not include supplemental vitamin D omit the largest source of vitamin D in supplement users, which accounts for a variable proportion of the population, notably older adults and young children [39,40,41]. The contribution made by this component of dietary exposure was illustrated by Whiting et al. [40], who reported that supplement users aged 6–79 years, representing 31% of the 2007–2009 Canadian Health Measures Survey-Cycle I sample, had higher 25(OH)D concentrations than non-users and a much lower prevalence of serum 25(OH)D < 50 nmol/L (19 vs. 37%). Intake estimates that ignore the contribution from supplements are not capable of assessing the risk to public health of low (and high) vitamin D intakes and are not fit for purpose in dietary modelling experiments for nutrient addition/fortification.

From a public health perspective, supplements are also a critical factor for risk managers, who, in developing public health nutrition strategies for vitamin D deficiency prevention, should consider that supplements are only effective in those who consume them. Notably, uptake is generally low among adolescents and young adults, who are at risk of vitamin D deficiency [40]. Therefore, recommending supplements is not an appropriate strategy to increase vitamin D intakes across the population distribution and reduce the prevalence of very low vitamin D status evident in many EU countries [14]. While we acknowledge the usefulness of supplements under medical supervision (e.g., as part of the management of medical conditions and during pregnancy and infancy, discussed above), public health strategy should be designed to meet the needs of the unsupervised majority, on an on-going basis. Thus, fortification is the preferred approach to bridge the gap between current vitamin D intakes and the lowest recommendation of 10 μg /day and to minimise the prevalence of serum 25(OH)D concentrations <30 nmol/L, without increasing the risk of chronic excessive intakes [43]. Among adults, the distribution of vitamin D intakes is typically heavily skewed to the left, with a small number of high-dose supplement users at the high end [39]. By increasing vitamin D in the usual food supply, intakes in the lower half of the distribution would increase, without increasing the proportion with intakes at or close to the tolerable upper intake levels [5].

#### 3.3.3. Evidence for food fortification

The evidence basis for this strategy is secure; systematic literature reviews of food-based RCTs have shown that food fortification increases serum 25(OH)D among children [44] and adults [45,46] and fresh evidence from Finland has demonstrated its effectiveness as a public health strategy for prevention of deficiency [47]. However, Black et al. [46] and others [42,48,49] have highlighted the problem of fortifying a single food item (such as milk), which is that similar to supplements, restriction of a fortification policy to a single sector (e.g., dairy) does not increase vitamin D intakes or status in non- or low consumers. Experimental evidence was provided in Denmark by Madsen et al. [50], who conducted a food-based wintertime RCT of vitamin D-fortified milk and bread in 201 families (*n* = 782 children and adults, aged 4–60 years). Median intakes of vitamin D were 2.2 and 9.6 μg/d, respectively in the groups randomized to vitamin D unfortified and fortified foods. At the end of the 6-months intervention, <1% of participants in the fortified group had serum 25(OH)D < 30 nmol/L, compared with 25% in the placebo group. The prevalence of 25(OH)D < 50 nmol/L was 16% and 65% in the fortified and unfortified groups, respectively [50]. With no evidence of an adverse effect, this is compelling evidence for efficacy of a multi-strand approach to low-dose fortification of commodity foods. In view of the evidence discussed earlier in relation to particular risks of vitamin D deficiency among ethnic minorities, dietary diversity must be accommodated for effective delivery of addition vitamin D to the population.

As stated above, access to quality assured food composition data for vitamin D, including both total vitamin D content and vitamins D_2_, D_3_ and 25-hydroxyvitamin D_3_ in the base diet, fortified foods and composite dishes is required to enable reliable calculations of vitamin D intakes as well as to facilitate predictive modelling for fortification strategy development [13]. To underpin its dietary research programme, ODIN compiled a specialized, quality assured, fully referenced food composition dataset of vitamin D, based on EuroFIR standards, using the FoodEXplorer™ tool to retrieve documented analytical data [51]. Data are stratified by European (8 countries, 981 data values) and United States origin (USDA, 1836 data values) and are classified according to LanguaL^TM^, FoodEX2 and ODIN classification systems, ranked according to defined quality criteria [51]. Incorporating up to date manufacturer’s data on fortification (where analytical data were not available) and a vitamin D-oriented food classification system, ODIN researchers simplified the analysis and dietary modelling of complex food consumption data (papers under review), which enabled us to incorporate new composition data coming from ODIN studies on low-fat cheese [52], eggs [53], UV-exposed mushrooms [54] and associated research on pork meat and beef [55,56,57], as well as existing data from voluntary fortification of milk, ready to eat breakfast cereals, and other products [39].

These studies [52,53,54,55,56,57] brought the food from “farm to fork”, demonstrating within the same study that animals fed with fortified feed could deliver additional vitamin D to consumers, safely and without compromising product quality. For example, Hayes et al. [53] showed that feeding hens additional vitamin D_3_ and/or 25(OH)D_3_ allowable under EU legislation produced eggs with increased vitamin D activity (~5 μg/egg), with no reduction in consumer acceptability. A winter-based RCT of older adults showed that the treatment group who were randomized to consume vitamin D-enriched eggs did not display the usual seasonal decline in serum 25(OH)D and had a zero prevalence <25 nmol/L [53]. In the control group, who consumed commercially available eggs, 25(OH)D decreased over 8 weeks of winter, and 22% were <25 nmol/L at endpoint [53]. No adverse effects were recorded in the treatment or control groups and there were no differences in serum total cholesterol between consumers of vitamin D-enriched eggs and controls [53]. Using a separate product, an ODIN RCT in post-menopausal women in Greece demonstrated that daily consumption of 60g of reduced-fat Gouda fortified with vitamin D_3_ increased serum 25(OH)D concentrations and prevented low 25(OH)D during wintertime among consumers [52].

Dietary modelling experiments undertaken in the project have been conducted according to current consumption practices across 10 nationally representative surveys in 4 countries and demonstrated the feasibility of achieving average intakes of ~10 μg/day vitamin D, without increasing the risk of excessive intakes (data under review). The ODIN hypothesis, that careful application of fortification strategies could safely increase intakes of vitamin D across the distribution and prevent low concentrations of 25(OH)D in populations subgroups, appears to be technically feasible. Further work is required in extending the diversity of foods under consideration, in conducting further dose-response experiments in various species (e.g., farmed fish and UV-irradiated commodities) and in conducting modelling experiments in more ethnically diverse food consumption surveys. Societal and economic benefits are currently hypothetical and these require further investigation.

## 4. Conclusions

In summary, selected outcomes from the ODIN project within the priority areas of vitamin D status, dietary requirements and intakes, as well as testing strategies for food fortification, have produced some outstanding new evidence, summarised in Box 2. The first internationally comparable dataset of vitamin D status showed that 13% of EU residents overall, across a latitude gradient of 35° N to 69° N, had serum 25(OH)D < 30 nmol/L and 40% were <50 nmol/L. The risk of very low vitamin D status was several-fold higher among persons of ethnic minority; however, further analysis of quality bio-banked sera would be required to improve the accuracy of these estimates. There is a need for strategic investment in quality, accessible surveillance and bio-banking systems among many countries in the Mediterranean, Central and Eastern European regions. Standardisation of population-based 25(OH)D measurements should be reported. Our dose-response RCT estimates of dietary requirements for vitamin D among under-researched life stage and population groups showed that to prevent seasonal 25(OH)D decreases in Northern Europe < 30nmol/L, vitamin D_3_ intakes of 8 and 13 μg/day are required by white children and adolescents and 20 and 30 μg/day, respectively, are needed to achieve ≥50 nmol/L. Among white women during pregnancy, 30 μg/day is required to prevent umbilical cord 25(OH)D, representing new-born vitamin D status, below 25 nmol/L. While 8 μg/day protected white women in Finland at the 30 nmol/L cut-off, 18 μg was needed by women of East African descent to prevent 25(OH)D decreasing below 30 nmol/L during wintertime. There is a pronounced variation between different ethnic groups in the 25(OH)D response to vitamin D_3_ supplementation. Replicate RCTs are needed in young children < 5 years and in school-age children, teens and pregnant women of ethnic minority. Using a series of animal-feeding trials, food production studies and food-based RCTs, followed by dietary modelling experiments, ODIN demonstrated that diverse fortification strategies could safely increase intakes of vitamin D across the distribution and prevent low concentrations of 25(OH)D in population subgroups. Further work in this area is required, in particular among ethnic minorities. Building on the solid technological platform provided by the ODIN consortium, implementation research is now warranted, to scale up interventions in real-world settings for vitamin D deficiency prevention.

Box 2 **Summary.**

**New data on vitamin D from ODIN and outstanding data requirements**

*Prevalence of vitamin D deficiency in Europe*

One in eight, or 13% of EU residents from 35° N to 69° N had serum 25(OH)D < 30 nmol/L and 40% were <50 nmol/L. Among whites, the highest risk was among adolescents. According to WHO criteria, persons of ethnic minority had a severe prevalence of very low vitamin D status, with estimates of 25(OH)D < 30 nmol/L between 30 and 60%, depending on ethnic group and country of residence.

*Data requirement*

Analysis of quality, well-characterised bio-banked sera of persons of ethnic minority and immigrant populations are required to improve vitamin D status estimates; this must be prioritised to identify policy and avoid persistent health inequalities.

*Dietary requirements for vitamin D during pregnancy, childhood and adolescence and impact of ethnicity*

For white children aged 4–8 years, 8 µg/day of vitamin D_3_ prevents 25(OH)D falling below 30 nmol/L during wintertime and 20 µg/day will bring 97.5% to 50 nmol/L.

For white teens aged 14–18 years, intakes of 13 µg/day are needed to prevent 25(OH)D from dropping <30 nmol/L during winter and at least 30 µg/day is required to achieve ≥50 nmol/L.

IPD analysis of 25(OH)D-vitamin D_3_ dose-response RCTs confirmed that 10, 13 and 26 µg/day would be required to maintain circulating 25(OH)D > 25, 30, and 50 nmol/L, respectively in almost all (i.e., at the level of a Recommended Dietary Allowance) white-skinned, non-pregnant persons.

The dose-response of 25(OH)D to vitamin D_3_ varies between persons of different ethnic backgrounds. While 8 μg/day protected white women in Finland at the 30 nmol/L cut-off, 18 μg/day was needed by women of East African descent to prevent 25(OH)D decreasing below 30 nmol/L during wintertime.

Among white women, 30 μg/d vitamin D_3_ safely maintains serum 25(OH)D concentrations ≥50 nmol/L during pregnancy at ~50° N, which in turn keeps 25(OH)D >25 nmol/L in 99% and ≥30 nmol/L in 95% of umbilical cord sera, preventing neonatal vitamin D deficiency.

*Data requirements*

Dose-response RCTs in young children (1–4 years); older children, teens, adults and pregnant women of different ethnic backgrounds.

*Effect of fortification in increasing population intakes and status of vitamin D*

Using a series of animal-feeding trials, food production studies and food-based RCTs, followed by dietary modelling experiments underpinned by a bespoke vitamin D food composition database, ODIN research shows that diverse fortification strategies could safely increase intakes of vitamin D and prevent low concentrations of 25(OH)D in population subgroups.

*Data requirements*

Building on this solid technological platform, implementation research is now warranted to scale up interventions in real-world settings for prevention of vitamin D deficiency.

## References

[1] Autier P., Mullie P., Macacu A., Dragomir M., Boniol M., Coppens K., Pizot C., Boniol M. (2017). Effect of vitamin D supplementation on non-skeletal disorders: A systematic review of meta-analyses and randomised trials. Lancet Diabetes Endocrinol..

[2] Ebeling P., Adler R., Jones G., Liberman U.A., Mazziotti G., Minisola S., Munns C., Napoli N., Pittas A., Giustina A. (2018). Management of endocrine disease: Therapeutics of vitamin D. Eur. J. Endocrinol..

[3] Manson J.E., Bassuk S.S., Lee I.M., Cook N.R., Albert M.A., Gordon D., Zaharris E., Macfadyen J.G., Danielson E., Lin J. (2012). The vitamin D and OmegA-3 TriaL (VITAL): Rationale and design of a large randomized controlled trial of vitamin D and marine omega-3 fatty acid supplements for the primary prevention of cancer and cardiovascular disease. Contemp. Clin. Trials.

[4] Pradhan A.D., Manson J.E. (2016). Update on the vitamin D and OmegA-3 trial (VITAL). J. Steroid Biochem. Mol. Biol..

[5] Ross A.C., Taylor C.L., Yaktine A.L., Del Valle H.B., Institute of Medicine (2011). Dietary Reference Intakes for Calcium and Vitamin D.

[6] Scientific Advisory Committee on Nutrition Report on Vitamin D and Health. http://www.sacn.gov.uk/pdfs/sacn_vitaminD_and_health_report_web.pdf.

[7] EFSA NDA Panel (EFSA Panel on Dietetic Products, Nutrition and Allergies) (2016). Scientific opinion on dietary reference values for vitamin D. EFSA J..

[8] NORDEN Nordic Nutrition Recommendations, 5th Edition (NNR5)—Vitamin D. http://www.slv.se/en-gb/Startpage-NNR/Public-consultation11/.

[9] German Nutrition Society (2012). New reference values for vitamin D. Ann. Nutr. Metab..

[10] Holick M.F., Binkley N.C., Bischoff-Ferrari H.A., Gordon C.M., Hanley D.A., Heaney R.P., Murad M.H., Weaver C.M. (2011). Evaluation, treatment, and prevention of vitamin D deficiency: An endocrine society clinical practice guideline. J. Clin. Endocrinol..

[11] Cashman K.D., Kiely M. (2011). Towards prevention of vitamin D deficiency and beyond: Knowledge gaps and research needs in vitamin D nutrition and public health. Br. J. Nutr..

[12] Cashman K.D., Kiely M. (2014). Recommended dietary intakes for vitamin D: Where do they come from, what do they achieve and how can we meet them?. J. Hum. Nut. Diet..

[13] Kiely M., Cashman K.D. (2015). The ODIN project: Development of food-based approaches for prevention of vitamin D deficiency throughout life. Nutr. Bull..

[14] Cashman K.D., Dowling K.G., Škrabáková Z., Gonzalez-Gross M., Valtueña J., De Henauw S., Moreno L., Damsgaard C.T., Michaelsen K.F., Molgaard C. (2016). Vitamin D deficiency in Europe: Pandemic?. Am. J. Clin. Nutr..

[15] Sarafin K., Durazo-Arvizu R., Tian L., Phinney K.W., Tai S., Camara J.E., Merkel J., Green E., Sempos C.T., Brooks S.P. (2015). Standardizing 25-hydroxyvitamin D values from the Canadian Health Measures Survey. Am. J. Clin. Nutr..

[16] Schleicher R.L., Sternberg M.R., Looker A.C., Yetley E.A., Lacher D.A., Sempos C.T., Taylor C.L., Durazo-Arvizu R.A., Maw K.L., Chaudhary-Webb M. (2016). National estimates of serum total 25-Hydroxyvitamin D and metabolite concentrations measured by Liquid Chromatography-Tandem Mass Spectrometry in the US population during 2007-2010. J. Nutr..

[17] De Benoist B., McLean E., Egli I., Cogswell M., World Health Organization (2008). Worldwide Prevalence of Anaemia 1993–2005: WHO Global Database on Anaemia.

[18] Sempos C.T., Vesper H.W., Phinney K.W., Thienpont L.M., Coates P.M. (2012). Vitamin D status as an international issue: National surveys and the problem of standardization. Scand. J. Clin. Lab. Invest. Suppl..

[19] Durazo-Arvizu R.A., Tian L., Brooks S.P.J., Sarafin K., Cashman K.D., Kiely M., Merkel J., Myers G.L., Coates P.M., Sempos C.T. (2017). The Vitamin D Standardization Program (VDSP) manual for retrospective laboratory standardization of serum 25-hydroxyvitamin D data. J. AOAC Int..

[20] Quraishi S.A., Camargo C.A., Manson J.E. (2016). Low vitamin D status in Europe: Moving from evidence to sound public health policies. Am. J. Clin. Nutr..

[21] Manios Y., Moschonis G., Lambrinou C.P., Tsoutsoulopoulou K., Binou P., Karachaliou A., Breidenassel C., Gonzalez-Gross M., Kiely M., Cashman K.D. (2018). A systematic review of vitamin D status in southern European countries. Eur. J. Nutr..

[22] Mortensen C., Damsgaard C.T., Hauger H., Ritz C., Lanham-New S.A., Smith T.J., Hennessy Á., Dowling K., Cashman K.D., Kiely M. (2016). Estimation of the dietary requirement for vitamin D in white children aged 4–8 y: A randomized, controlled, dose-response trial. Am. J. Clin. Nutr..

[23] Smith T.J., Tripkovic L., Damsgaard C.T., Molgaard C., Ritz C., Wilson-Barnes S.L., Dowling K.G., Hennessy Á., Cashman K.D., Kiely M. (2016). Estimation of the dietary requirement for vitamin D in adolescents aged 14–18 y: A dose-response, double-blind, randomized placebo-controlled trial. Am. J. Clin. Nutr..

[24] Adebayo F.A., Itkonen S.T., Ohman T., Skaffari E., Saarnio E.M., Erkkola M., Cashman K.D., Lamberg-Allardt C. (2018). Vitamin D intake, serum 25-hydroxyvitamin D status and response to moderate vitamin D3 supplementation: A randomised controlled trial in East African and Finnish women. Br. J. Nutr..

[25] Cashman K.D., Ritz C., Adebayo F.A., Dowling K.G., Itkonen S.T., Öhman T., Skaffari E., Saarnio E.M., Kiely M., Lamberg-Allardt C. (2018). Differences in the dietary requirement for vitamin D among Caucasian and East African women at Northern latitude. Eur. J. Nutr..

[26] O’Callaghan K.M., Hennessy Á., Hull G.L., Healy K., Ritz C., Kenny L.C., Cashman K.D., Kiely M.E. (2018). Estimation of the maternal vitamin D intake that maintains circulating 25-hydroxyvitamin D in late gestation at a concentration sufficient to keep umbilical cord sera ≥25–30 nmol/L: A dose-response, double-blind, randomized placebo-controlled trial in pregnant women at northern latitude. Am. J. Clin. Nutr..

[27] Ohlund I., Lind T., Hernell O., Silfverdal S.A., Karlsland Åkeson P. (2017). Increased vitamin D intake differentiated according to skin color is needed to meet requirements in young Swedish children during winter: A double-blind randomized clinical trial. Am. J. Clin. Nutr..

[28] Gallagher J.C., Jindal P.S., Smith L.M. (2014). Vitamin D supplementation in young White and African American women. J. Bone. Miner. Res..

[29] O’Callaghan K.M., Kiely M.E. (2018). Ethnic disparities in the dietary requirement for vitamin D during pregnancy: Considerations for nutrition policy and research. Proc. Nutr. Soc..

[30] Kiely M., Hemmingway A., O’Callaghan K.M. (2017). Vitamin D in pregnancy: Current perspectives and future directions. Ther. Adv. Musculoskelet. Dis..

[31] O’Callaghan K.M., Kiely M. (2018). Systematic review of vitamin D and hypertensive disorders of pregnancy. Nutrients.

[32] Munns C.F., Shaw N., Kiely M., Specker B.L., Thacher T.D., Ozono K., Michigami T., Tiosano D., Mughal M.Z., Mäkitie O. (2016). Global consensus recommendations on prevention and management of nutritional rickets. J. Clin. Endocrinol. Metab..

[33] Kiely M., O’Donovan S.M., Kenny L.C., Hourihane J.O., Irvine A.D., Murray D.M. (2017). Vitamin D metabolite concentrations in umbilical cord blood serum and associations with clinical characteristics in a large prospective mother-infant cohort in Ireland. J. Steroid Biochem. Mol. Biol..

[34] Cashman K.D., Ritz C., Kiely M. (2017). Improved dietary guidelines for vitamin D: Application of Individual Participant Data (IPD)-level meta-regression analyses. Nutrients.

[35] O’Neill C.M., Kazantzidis A., Ryan M.J., Barber N., Sempos C.T., Durazo-Arvizu R.A., Jorde R., Grimnes G., Eiriksdottir G., Gudnason V. (2016). Seasonal changes in vitamin D-effective UVB availability in Europe and associations with population serum 25-Hydroxyvitamin D. Nutrients.

[36] Roseland J.M., Phillipis K.M., Patterson K.Y., Pehrsson P.R., Taylor C.L., Feldman D., Pike J.W., Bouillon R., Giovannucci E., Goltzman D., Hewison M. (2018). Vitamin D in foods: An evolution of knowledge. Vitamin D.

[37] Whiting S.J., Calvo M.S., Feldman D., Pike J.W., Bouillon R., Giovannucci E., Goltzman D., Hewison M. (2018). Vitamin D fortification and supplementation policies to correct vitamin D insufficiency/deficiency globally. Vitamin D.

[38] Roman Viñas B., Ribas Barba L., Ngo J., Gurinovic M., Novakovic R., Cavelaars A., de Groot L.C., van’t Veer P., Matthys C., Serra Majem L. (2011). Projected prevalence of inadequate nutrient intakes in Europe. Ann. Nutr. Metab..

[39] Black L.J., Walton J., Flynn A., Cashman K.D., Kiely M. (2015). Small increments in vitamin D intake by Irish adults over a decade sShow that strategic initiatives to fortify the food supply are needed. J. Nutr..

[40] Whiting S.J., Langlois K.A., Vatanparast H., Greene-Finestone L.S. (2011). The vitamin D status of Canadians relative to the 2011 Dietary Reference Intakes: An examination in children and adults with and without supplement use. Am. J. Clin. Nutr..

[41] Fulgoni V.L., Keast D.R., Bailey R.L., Dwyer J. (2011). Foods, fortificants, and supplements: Where do Americans get their nutrients?. J. Nutr..

[42] Kiely M., Black L.J. (2012). Dietary strategies to maintain adequacy of circulating 25-hydroxyvitamin D concentrations. Scand. J. Clin. Lab. Invest. Suppl..

[43] Cashman K.D., Kiely M., Feldman D., Pike J.W., Bouillon R., Giovannucci E., Goltzman D., Hewison M. (2018). Vitamin D and food fortification. Vitamin D.

[44] Brett N.R., Gharibeh N., Weiler H.A. (2018). Effect of vitamin D supplementation, food fortification, or bolus injection on vitamin D status in children aged 2–18 years: A meta-analysis. Adv. Nutr..

[45] O’Donnell S., Cranney A., Horsley T., Weiler H.A., Atkinson S.A., Hanley D.A., Ooi D.S., Ward L., Barrowman N., Fang M. (2008). Efficacy of food fortification on serum 25-hydroxyvitamin D concentrations: Systematic review. Am. J. Clin. Nutr..

[46] Black L.J., Seamans K.M., Cashman K.D., Kiely M. (2012). An updated systematic review and meta-analysis of the efficacy of vitamin D food fortification. J. Nutr..

[47] Jääskeläinen T., Itkonen S.T., Lundqvist A., Erkkola M., Koskela T., Lakkala K., Dowling K.G., Hull G.L., Kröger H., Karppinen J. (2017). The positive impact of general vitamin D food fortification policy on vitamin D status in a representative adult Finnish population: Evidence from an 11-y follow-up based on standardized 25-hydroxyvitamin D data. Am. J. Clin. Nutr..

[48] Cashman K.D., Kiely M. (2016). Tackling inadequate vitamin D intakes within the population: Fortification of dairy products with vitamin D may not be enough. Endocrine.

[49] Calvo M.S., Whiting S.J. (2006). Public health strategies to overcome barriers to optimal vitamin D status in populations with special needs. J. Nutr..

[50] Madsen K.H., Rasmussen L.B., Andersen R., Molgaard C., Jakobsen J., Bjerrum P.J., Andersen E.W., Mejborn H., Tetens I. (2013). Randomized controlled trial of the effects of vitamin D-fortified milk and bread on serum 25-hydroxyvitamin D concentrations in families in Denmark during winter: The vitamin D study. Am. J. Clin. Nutr..

[51] Milešević J., Samaniego L., Kiely M., Glibetić M., Roe M., Finglas P. (2018). Specialized food composition dataset for vitamin D content in foods based on European standards: Application to dietary intake assessment. Food Chem..

[52] Manios Y., Moschonis G., Mavrogianni C., van den Heuvel E., Singh-Povel C.M., Kiely M., Cashman K.D. (2017). Reduced-fat Gouda-type cheese enriched with vitamin D3 effectively prevents vitamin D deficiency during winter months in postmenopausal women in Greece. Eur. J. Nutr..

[53] Hayes A., Duffy S., O’Grady M., Jakobsen J., Galvin K., Teahan-Dillon J., Kerry J., Kelly A., O’Doherty J., Higgins S. (2016). Vitamin D-enhanced eggs are protective of wintertime serum 25-hydroxyvitamin D in a randomized controlled trial of adults. Am. J. Clin. Nutr..

[54] Cashman K.D., Kiely M., Seamans K.M., Urbain P. (2016). Effect of ultraviolet light-exposed mushrooms on vitamin D status: Liquid Chromatography-Tandem Mass Spectrometry Reanalysis of biobanked sera from a randomized controlled trial and a systematic review plus Meta-Analysis. J. Nutr..

[55] Duffy S.K., Kelly A.K., Rajauria G., Jakobsen J., Clarke L.C., Monahan F.J., Dowling K.G., Hull G., Galvin K., Cashman K.D. (2018). The use of synthetic and natural vitamin D sources in pig diets to improve meat quality and vitamin D content. Meat Sci..

[56] Duffy S.K., O’ Doherty J.V., Rajauria G., Clarke L.C., Hayes A., Dowling K.G., O’Grady M.N., Kerry J.P., Jakobsen J., Cashman K.D. (2018). Vitamin D-biofortified beef: A comparison of cholecalciferol with synthetic versus UVB-mushroom-derived ergosterol as feed source. Food Chem..

[57] Duffy S.K., O’ Doherty J.V., Rajauria G., Clarke L.C., Cashman K.D., Hayes A., O’Grady M.N., Kerry J.P., Kelly A.K. (2017). Cholecalciferol supplementation of heifer diets increases beef vitamin D concentration and improves beef tenderness. Meat Sci..

